# Recent Advances in Thermal Interface Materials for Thermal Management of High-Power Electronics

**DOI:** 10.3390/nano12193365

**Published:** 2022-09-27

**Authors:** Wenkui Xing, Yue Xu, Chengyi Song, Tao Deng

**Affiliations:** 1 The State Key Laboratory of Metal Matrix Composites, School of Materials Science and Engineering, Shanghai Jiao Tong University, 800 Dong Chuan Road, Shanghai 200240, China; 2 Shanghai Key Laboratory of Hydrogen Science & Center of Hydrogen Science, Shanghai Jiao Tong University, Shanghai 200240, China

**Keywords:** thermal interface materials, thermal conductivity, high-power electronics, interfacial thermal resistance, thermal management

## Abstract

With the increased level of integration and miniaturization of modern electronics, high-power density electronics require efficient heat dissipation per unit area. To improve the heat dissipation capability of high-power electronic systems, advanced thermal interface materials (TIMs) with high thermal conductivity and low interfacial thermal resistance are urgently needed in the structural design of advanced electronics. Metal-, carbon- and polymer-based TIMs can reach high thermal conductivity and are promising for heat dissipation in high-power electronics. This review article introduces the heat dissipation models, classification, performances and fabrication methods of advanced TIMs, and provides a summary of the recent research status and developing trends of micro- and nanoscale TIMs used for heat dissipation in high-power electronics.

## 1. Introduction

Due to the highly integrated and hierarchical structure of high-power density electronics, there is tremendous localized thermal energy generated per unit time, which indicates advanced thermal management is necessary to reach higher stipulations of heat dissipation [1,2,3,4]. Effective thermal management could guarantee normal operation and efficient device performance, especially for high-power electronics with heat fluxes of 150–200 W·cm^−2^ and pulsed transient heat loads with fluxes of more than 400 W·cm^−2^ [5], such as automotive vehicles [6], defense electronics [7], aerospace [8] and computer processors [9]. Structural cooling systems such as microchannels for liquid cooling have been widely applied in electronics for effective heat dissipation [10,11]. The convection flow in microchannels contributes to the cooling process by removing the generated heat from electronics. For more integrated equipment with a smaller size and less operation space, air cooling can also be used in the high-power electronic system by adding cooling sinks into the electronic devices [12].

Usually, the contact between the chip and the cooling sink is not ideal due to the roughness of both materials at the microscale. Therefore, air with low thermal conductivity of only 0.024 W·m^−1^·K^−1^ always fills the spaces between the interfaces, resulting in high thermal contact resistance. Therefore, the implementation of advanced thermal interface materials (TIMs) plays a critical role in the effective thermal management of high-power electronics. TIMs are used to improve heat dissipation efficiency in electronic devices and are usually positioned between the chip and heat sink. They can fit well with the interfaces and eliminate air voids by filling the air gap between the chip and heat sink. Therefore, high thermal conductive TIMs should enhance heat transfer and reduce the thermal contact resistance. Additionally, TIMs require appropriate thermal expansion to match the working chips to minimize interfacial thermal resistance. As a result, the development of advanced TIMs and improvement in their thermal conductivity are of vital importance in the manufacture process of high-performance electronic devices. There are numerous types of TIMs commercially available or under development, including thermal grease/paste, carbon-based materials, phase change materials and filled polymers, which are suitable for employment in various kinds of electronic devices [13]. Low interfacial thermal resistance and high thermal conductivity, good adhesion and conformability are the general requirements for TIMs. Furthermore, TIMs should also have long-term stability, be nontoxic and not spill to keep pace with high-power electronic system technologies.

**Figure 1:**
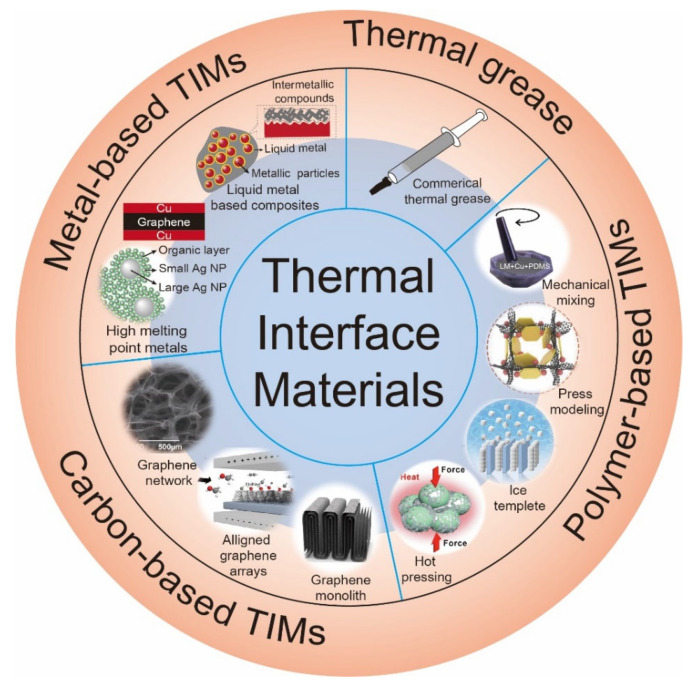
Schematic summary of advanced TIMs for the heat dissipation of high-power electronics.

This review will focus on the heat dissipation model of TIMs used in high-power electronics, as well as different types of advanced TIMs, and present new insights and challenges for the application of advanced TIMs in high-power electronic devices (Figure 1).

## 2. Heat Dissipation Model of TIMs Used in High-Power Electronics

Decreasing interfacial thermal resistance and enhancing the intrinsic thermal conductivity of TIMs are both effective methods to improve heat dissipation efficiency. Most TIMs are composites; therefore, it is of great significance to understand their heat conduction and dissipation models.

### 2.1. Thermal Resistance Models

During the heat dissipation of high-power electronic devices, heat flows from the high-temperature part to the low-temperature part. Thermal contact resistance (also known as Kapitza resistance) occurs between the solid substrate and TIMs, which impedes the propagation of heat flow. Apart from that, filler–filler and filler–matrix thermal contact resistance inside TIMs should also be considered for the whole system. The nanoscale fillers in the TIMs would introduce more interfaces, which would cause intense phonon scattering and impede the process of heat transfer. As a result, the control and further study of thermal contact resistance are of great significance in the development of advanced TIMs.

For metal-based TIMs, the thermal contact resistance at metallic–metallic interfaces is mainly caused by the scattering of electrons influenced by lattice mismatching and defects. For other metallic–nonmetallic interfaces, both phonons and electrons could be the carriers for heat conduction. Zhou and Li [14] proposed a metallic–nonmetallic interfacial heat transfer model, which pointed out that heat could transfer through the phonon–phonon coupling, phonon–electron coupling, and electron–electron coupling processes between metallic particles, the polymer matrix and nonmetallic particles. The degree of competition between these three kinds of transportation processes would determine the value of the thermal contact resistance.

Recently, a variety of methods have been introduced for predicting thermal contact resistance, including theoretical models, atomistic methods and experimental measurement methods [15,16]. Regarding the basic theoretical models, Swartz and Pohl [17] introduced the acoustic mismatch model (AMM) and the diffuse mismatch model (DMM), both of which consider that the interface at both sides of two materials has continuity. For the AMM, the incident phonons conducting heat can be treated as plane waves, and possible scattering can be ignored, which mean this model only matches well with actual situations at low temperatures (T < 10 K). In contrast to the AMM, the DMM considers that all the incident phonons are completely scattered, and can predict thermal contact resistance at about T > 15 K, but it is not applicable at higher temperatures. More developments based on these two models have also been made. Prasher [18] modified the traditional AMM and considered the influence of the van der Waals force between the nanofiller–nanofiller and nanofiller–matrix. Hopkins and Norris [19] proposed a modified joint frequency diffuse mismatch model (JFDMM) based on the DMM, which includes the effect of inelastic scattering of phonons. Atomistic methods, such as the Boltzmann equation [20], molecular dynamics (MD) [21] and Green’s function [22], can also be used to study interfacial thermal transportation at the atomic scale by simulating the motivation of the hot carriers, and use basic potential functions to calculate the thermal contact resistance between different materials.

**Figure 2:**
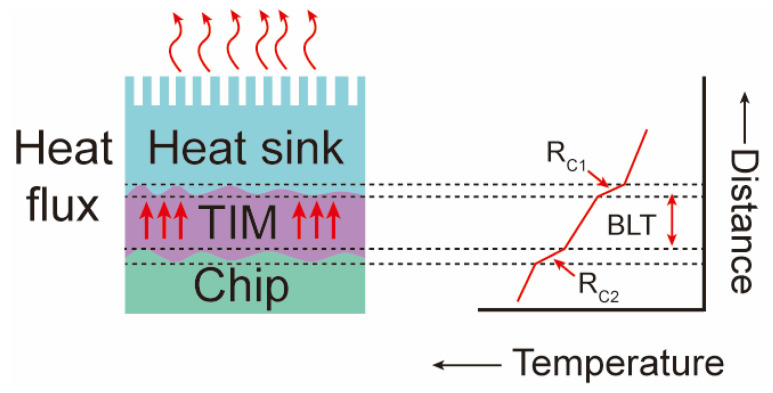
Schematic diagram of heat dissipation and interfacial thermal resistance in high-power electronics.

According to the schematic diagram of high-power electronics’ heat dissipation in Figure 2, the interfacial thermal resistance of TIMs (R_TIM_) can be shown as follows [23]:

(1) $$R_{\mathrm{TIM}}=\frac{\mathrm{BLT}}{k_{\mathrm{TIM}}}{\;+\;R}_{C1}{\;+\;R}_{C2\;},$$

where BLT refers to the bond line thickness of TIMs, k_TIM_ refers to the thermal conductivity of TIMs, R_C1_ is the thermal contact resistance between the TIM and the heat sink, and R_C2_ is the thermal contact resistance between the TIM and the chip. This formula shows that TIMs with lower BLT and higher thermal conductivity are ideal for achieving lower interfacial thermal resistance.

When calculating BLT, a suitable calculation model should be selected according to the physical state of TIMs. Prasher’s scaling-bulk model [24] showed BLT is primarily related with the yield stress of the polymer matrix *τ_y_*. The formula can be given as [25]:

(2) $$\mathrm{BLT}=\frac{2}{3}r\left(\frac{\tau_{y}}{P}\right)\;+\;{\left(\frac{\mathrm{cr}}{1.5}\right)}^{0.188}d^{0.811}\times {\left(\frac{\tau_{y}}{P}\right)}^{0.188},$$

where *r* is the substrate radius, P is the applied pressure, c is a constant number, and d is the particle diameter. Hu [26] introduced a two-medium model of fluid viscidity and filler particle contact interactions, and found that BLT is highly related to the particle contact interactions when the thickness of the TIM decreases to a particular value.

The properties of the contact surface, such as the surface roughness, thermal conductivity of the contacting materials, hardness and contact pressure, can influence the thermal contact resistance [27]. The sum of the thermal contact resistance R_c_ can be defined as:

(3) $$R_{C}{\;=R}_{c1}{\;+\;R}_{c2}=\left(\frac{\sigma_{1}{\;+\;\sigma }_{2}}{{2k}_{\mathrm{TIM}}}\right)\left(\frac{A_{\mathrm{nominal}}}{A_{\mathrm{real}}}\right),$$

where $\sigma_{1}$ and $\sigma_{2}$ are the roughness of the contact interfaces of the heat sink and chip materials, and A_nominal_ and A_real_ are the nominal and actual microscopic area of the two contact interfaces, respectively.

For solid-like TIMs, plastic deformation can be used to estimate the ratio of the nominal and actual microscopic area. Thus, the total thermal contact resistance R_c_ can be further defined as [28]:

(4) $$\frac{1}{\mathrm{Rc}}=\frac{2{.5k}_{1}k_{2}}{k_{1}{\;+\;k}_{2}}\left(\frac{m}{\sqrt{\sigma_{1}{\;+\;\sigma }_{2}}}\right){\left(\frac{P}{H}\right)}^{0.95},\;$$

where k_1_ and k_2_ are the thermal conductivity of the heat sink and chip materials, m is the effective absolute mean asperity slope, P is the contact pressure, and *H* is the surface microhardness. The increase in contact pressure makes the surfaces fit more tightly, increases the contact area and enhances the heat transfer at the surface.

For liquid-like TIMs, the models based on the plastic deformation mentioned above are not applicable since these TIMs have high fluidity and will adhere to the substrate with no plastic deformation. In this case, A_nominal_ and A_real_ need to be calculated by the difference in surface energy resulting from different adhesions of fluidic TIMs to the substrate surface [27].

### 2.2. Thermal Conductivity Models

The main heat conduct carriers are electrons in the case of metals above room temperature, while the main carriers are phonons in the case of nonmetal. From a microscale point of view, the thermal resistance at the interfaces between the matrix and fillers inside the composite materials is attributed to the difference in electron or phonon vibration properties [23]. Electron and phonon scattering will occur while heat carriers are passing through the contact interface; therefore, the large difference in carrier density between two sides can cause a dramatically decrease in the thermal conductivity of composites, and thus impedes the heat transfer process.

Since most TIMs are composites, both phonons and electrons can be the carriers in conducting heat, and various models are used to calculate their thermal conductivities. For metals and alloys, the electronic transport efficiency is high and is involved in the conduction of thermal and electrical energy. The Wiedemann–Franz law [29] indicates that thermal conductivity is proportional to the electrical conductivity. The scattering and electron–electron interactions at the surface will also impact the thermal conductivity of metals and alloys.

In addition to metal-based TIMs, composite-based TIMs cover a wide range, and plenty of models can be used to calculate their thermal conductivity.

One of the typical composite-based TIMs is composed of an organic matrix and highly thermal conductive fillers. Table 1 provides a brief summary of the typical models used in calculating the thermal conductivity of composite materials, among which the Maxwell model and Bruggeman model are the most used ones. The Maxwell model [30,31] can characterize polymer-based composites with randomly dispersed sphere-shaped fillers at a low volume fraction, which does not include the interfacial thermal resistance between matrix and fillers. Based on the Maxwell model, the Hasselman and Johnson model [32] considers the influence of the filler size and interfacial thermal resistance between the filler and matrix to calculate the thermal conductivity. This model uses the reciprocal of the interfacial thermal conductance to represent interfacial thermal resistance and is widely used in predicting the thermal conductivity of metal-based and carbon-based composites. The phonon–electron interaction at the filler–matrix interfaces will increase the interfacial thermal resistance and make it nonnegligible. Molina et al. [33] employed the Hasselman–Johnson model to estimate the effect of porosity on the thermal conductivity of Al-12 wt % Si–graphite composites. Caccia and coworkers [34] improved the interfacial thermal conductance of Al–diamond composites via surface modification of diamond particles. They successfully utilized Maxwell and Hasselman–Johnson models to predict the thermal conductivity of the samples. In addition, Prieto and his coworkers [35] investigated the influence of the amount and particle size of SiC on graphite flakes–SiC particles–metal composites, whose theoretical thermal conductivity was predicted by the Hasselman–Johnson model. On the contrary, the Bruggeman model [36,37] uses mean field theory to consider the interactions between matrices and fillers. Furthermore, it can be also applied at relatively high volume fractions [38]. There are also more comprehensive models that consider the type, shape and size distribution of fillers, as can be seen in Table 1, and these models can all be applied in TIMs with nanoscale fillers according to the previous works [30,34,39,40,41].

As for the other inorganic TIMs composed of carbon or other cubic III–V compounds, phonon transport is the main heat conduction path. The slack model [42] can be used to explain the high thermal conductivity of graphite, diamond-like [43] and boron carbide crystal structures. For these inorganic TIMs, with a low atomic mass of constituent elements, high bonding strength, simple crystal structure and less phonon collision, the heat transfer will be largely enhanced.

One method used to improve thermal conductivity [44] includes increasing the number of fillers to induce thermal percolation [23], which can create a network in the material for thermal conduction and form effective heat transfer paths. Another effective way is to reduce interfacial thermal resistance by surface modification of fillers. The formation of an intermetallic alloy with high thermal conductivity can accelerate the heat transfer rate in metal-based TIMs. If fillers have thermal conducting anisotropy, uniform alignment will increase the thermal conductivity in a specific direction. In the next section, we will introduce different types of TIMs with high thermal conductivity that could be used in high-power electronic devices.

## 3. Types of Advanced Thermal Interface Materials for High-Power Electronics

### 3.1. Thermal Grease

Thermal greases are the first generation of TIMs and the most widely used in electronic equipment before 1990 [54]. They have a paste-like ability and low BLT, ranging from about 0.5 to 1 mm, which helps decrease the thermal resistance between the interfaces. Silicone or hydrocarbon oils are usually used as matrix materials for their good combination of mobility and viscosity, and can fill in the air gap more precisely. Polyethylene glycol (PEG), sodium silicate [55] and paraffin wax are also used as matrix materials due to their unique abilities, such as their better wetting ability and higher thermal conductivity, and they can also better accommodate thermal conductive fillers. Inorganic fillers, such as aluminum oxide (Al_2_O_3_), zinc oxide (ZnO) and silicon carbide (SiC), and metallic fillers, such as silver (Ag) and copper (Cu), can be mixed into the matrix and make the thermal grease highly thermal conductive. Naghibi and his colleagues [56] have proposed a kind of advanced thermal grease based on mineral oil and graphene as a filler, which can reach a thermal conductivity of 7.1 W·m^−1^·K^−1^. For a metal-based filler, Uppal [57] introduced liquid metal as fillers and microscale Ag needles to connect the liquid metal droplets. The multiphase silicone-oil-based thermal grease can achieve a thermal conductivity of up to 17 W·m^−1^·K^−1^.

However, most of the reported thermal greases have relatively low thermal conductivities, and are not suitable for heat dissipation of high-power devices. Additionally, thermal greases face serious failure mechanisms after a long period of use. When the two surfaces face thermomechanical stresses, the thermal grease is prone to pumping out from the gap due to its high fluidity. Additionally, at high temperatures, the filled particles and the matrix will separate, resulting in losing the ability of heat conduction [58]. High-power electronics are sophisticated and require a high-performance heat dissipation rate during use; therefore, thermal greases still need further research development to achieve better performances.

### 3.2. Metal-Based Thermal Interface Materials

Recently, advanced metal-based TIMs have obtained extensive attention in thermal management for high-power electronics due to their outstanding performances, especially their high thermal conductivities [59,60,61,62,63]. Thermal conductivity is one of the most crucial criteria for advanced TIMs since it can represent the heat dissipation capability of high-power circuit electronic systems.

**Figure 3:**
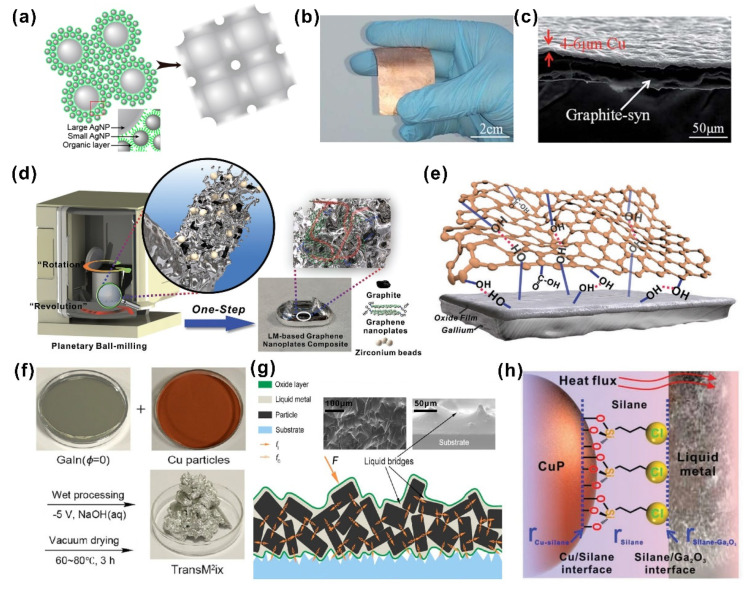
(**a**) The bimodal silver nanoparticle pastes via a sintering approach (Reprinted/adapted with permission from Ref. [65]. 2015, the American Chemical Society). (**b**) The picture of copper–graphite–copper (Cu–G–Cu) sandwich-structured TIMs. (**c**) The cross-sectional SEM image of the Cu–G–Cu sandwich ((**b**,**c**) reprinted/adapted with permission from Ref. [59]. 2016, Royal Society of Chemistry). (**d**) Schematic illustration of the facile one-step ball milling procedure to construct 3D thermal conductive graphene network. (**e**) The hydrogen bonding interaction between graphene nanoplates and liquid metal oxide ((**d**,**e**) reprinted/adapted with permission from Ref. [61]. 2021, Wiley). (**f**) The fabrication of obtained “TransM^2^ixes” liquid metal amalgams. (**g**) Schematic illustration of efficient thermal conduction paths constructed by the compact stacking of copper particles within liquid metal nanobridges ((**f**,**g**) reprinted/adapted with permission from Ref. [72]. 2017, the American Chemical Society). (**h**) The strong electrostatic interactions between CPTES covering the surface of copper particles with the freshly generated gallium oxide layer inside liquid metals. (Reprinted/adapted with permission from Ref. [62]. 2021, Wiley).

Advanced metal-based TIMs are generally composed of a metal matrix and highly thermally conductive fillers. The resultant composites with high thermal conductivity could be employed for heat dissipation of high-power electronics. High-melting-point metals such as silver [64,65,66], copper [59,62,67] and aluminum [68,69] were firstly explored and used in thermal management owing to their excellent heat dissipation properties. For example, Li et al. [65] introduced bimodal sintered silver nanoparticle pastes as TIMs with significant improvements in thermal and mechanical performance via a simple mixing and sintering approach. The resultant silver nanoparticle paste was prepared by sintering two sizes of silver nanoparticles (10 and 50 nm in diameter) at 250 °C for 30 min, where smaller particles acted as fillers to weld all of the particles together at a lower temperature, and large particles were utilized as frames to diminish crystallographic defects and stabilize the resultant sintered structure, as shown in Figure 3a. After sintering, it exhibited the unique compact three-dimensional network structures, leading to a superb thermal conductivity of 278.5 W·m^−1^·K^−1^, which could be assumed to be due to the generation of high-density coherent twins inside. The bimodal sintered silver nanoparticle paste could display a great heat dissipation capacity in high-power circuit electronics.

Jiang and his colleagues [59] proposed a fresh scenario to achieve high thermal conduction performance by fabricating copper–graphite–copper (Cu–G–Cu) sandwich-structured TIMs. The Cu–G–Cu sandwich with a metallic luster was fabricated through electroplating the copper layer on both sides of the graphite sheets; a picture is shown in Figure 3b. The cross-sectional SEM image of the Cu–G–Cu sandwich reveals that the thickness of the electroplated copper layer was 4–6 μm (Figure 3c). A distinguished thermal conductivity of 526–626 W·m^−1^·K^−1^ was eventually achieved, and low thermal contact resistance was demonstrated by a tight binding between copper layers and graphite sheets. Flexible mechanical properties and good weldability also allow it to cool electronic power devices.

These metal-based TIMs exhibit high thermal conductivity and distinguished heat dissipation performances, although air gaps may occur between heat sinks and electronics because of the intrinsic hard characteristics of high-melting-point metallic materials, and thus large interfacial thermal resistance is probably produced. Therefore, it could be challenge to use such metal-based TIMs in the thermal management of high-power electronics.

Liquid metal, as a class of low-melting-point metal with high thermal conductivity, and including materials such as gallium (Ga), eutectic gallium–indium alloy (EGaIn), eutectic gallium–indium–tin alloy (GaInSn) and other Ga-based metals, has gradually evolved into a new generation of promising advanced TIMs [62,63,70]. The good fluidity of liquid metals allows them to fill air cavities between the surfaces of two materials, thus reducing the interfacial thermal resistance; however, on the other hand, the nature of the fluidity of liquid metals may result in leakage during application, which contaminates high-power electronics [67]. Highly thermally conductive fillers, such as graphene [61,70], diamond [63,71], copper [72,73], tungsten [60] and boron nitride [74], are often blended into liquid metals to improve the adhesion and wettability of liquid metal composites, as well as to further enhance their thermal performance in the thermal management of high-power electronics.

Two-dimensional materials such as graphene, graphite and boron nitride and so on have been often employed as thermally conductive fillers to modify liquid metals. Wang et al. [70] reported a general approach (mechanical mixing, followed by oscillating ball milling) to produce composites of liquid metal and nonmetallic fillers with size-dependent and oxide-assisted effects. Mechanical mixing ahead of the ball milling was necessary to generate enough gallium oxide layers for the subsequent processing. They also stated that there was a size requirement for nonmetallic particles in their method: for instance, the GO flake filler needs to be larger than 10 μm. The engendered rGO–liquid gallium putty with porous microstructures exhibited a high thermal conductivity of 126 W·m^−1^·K^−1^ parallel to the sheet and 10.5 W·m^−1^·K^−1^ perpendicular to the sheet. This anisotropic mismatched 2D thermal conduction may invoke hindrances in the heat dissipation of high-power electronics. In our previous work [61], we adopted a facile one-step ball milling procedure to construct a 3D thermal conductive graphene network, leading to an enhanced 3D thermal conductivity of 44.6 W·m^−1^·K^−1^ and an electrical conductivity of 8.3 S·µm^−1^, as illustrated in Figure 3d. Such a one-step ball milling mechanochemistry process could eliminate the impediments on the size of the filler and accomplish the exfoliation of graphite by introducing carboxyl and hydroxyl functional groups at the edges of graphene nanoplates at the same time (Figure 3e). This method enables the stable dispersion of Ga and graphene nanoplates through the hydrogen bonding interaction and formation of a 3D network of thermal conductive pathways inside the composite. The improvement in thermal conductivity and wettability lead to a low interfacial thermal resistance of liquid metal composites when they come into contact with high-power circuit electronics and heat sinks as a TIM. Furthermore, we have demonstrated that they can be manipulated under external electric and magnetic fields, and thus present promising prospects for applications regarding field-driven systems.

Metallic particles such as copper [73], tungsten [60] and iron [75] have been regularly used to boost the thermal properties and wettability of liquid metal composites. Tang et al. [72] found that copper particles could be incorporated into a liquid metal matrix via an electrical polarization technique under a NaOH solution (Figure 3f). The obtained “TransM^2^ixes” liquid metal amalgams showed a thermal conductivity of 50 W·m^−1^·K^−1^ and an electrical conductivity of 6 × 10^6^ S·m^−1^, as well as flexible mechanical properties. The enhanced thermal performance could be ascribed to the generation of CuGa_2_ alloys during intermetallic alloying of liquid metal and copper. As revealed by Figure 3g, efficient thermal conduction paths were constructed by the compact stacking of copper particles within liquid metal nanobridges, which disclosed its capability for heat dissipation as advanced thermal management materials. Liquid metal amalgams, however, would gradually turn rigid and hard due to the in situ alloying of CuGa_2_ intermetallic compounds and continuous consumption of the liquid metal matrix. The lack of durability and flexibility inhibited its further development in high-power electronic systems. Recently [62], we established an interfacial engineering method in which 3-chloropropyltriethoxysilane (CPTES) was implemented as a thermomolecular linker for copper nanoparticles to improve the thermal performance and stability of liquid metal composites. The CPTES covering the surface of copper particles was linked to the freshly generated gallium oxide layer inside liquid metals by the strong electrostatic interactions, forming a chemical reaction barrier between the copper nanoparticle and liquid metal, which effectively suppressed further alloying and hardening of copper (Figure 3h). Other surface thermal linkers, such as thiol (-SH) and amino (-NH_2_) end silanes, were also employed for comparison with CPTES (-Cl). Since CPTES has a stronger electronegativity with the chlorine (-Cl)-terminated group, the electrostatic interaction strength of the -Cl group with gallium oxide was the strongest, which is possibly why the thermal conduction and chemical stability were enhanced. The use of an advanced TIM led to a thermal conductivity of 65 W·m^−1^·K^−1^ and long-term stability, and inherent thermal properties could be tailored for different electronic device scenarios. We have successfully investigated its effectiveness in the heat dissipation of high-power CPU electronic chips.

### 3.3. Carbon-Based Thermal Interface Materials

In recent decades, there has been an exciting development of carbon-based TIMs due to the ultrahigh theoretical thermal conductivity of carbon-based nanomaterials [76,77,78,79,80]. Carbon-based TIMs generally include materials related to graphene (3500−5300 W·m^−1^·K^−1^) [81], carbon nanotubes (CNTs, higher than 3000 W·m^−1^·K^−1^) [82], graphite, reduced graphene oxide (rGO), graphene oxide (GO) and single-walled carbon nanotubes (SWCNTs). Carbon-based TIMs with high thermal conductivity and low thermal contact resistance with other metallic substrates have been employed extensively to meet the demand for cooling high-power electronics [78,82,83,84].

**Figure 4:**
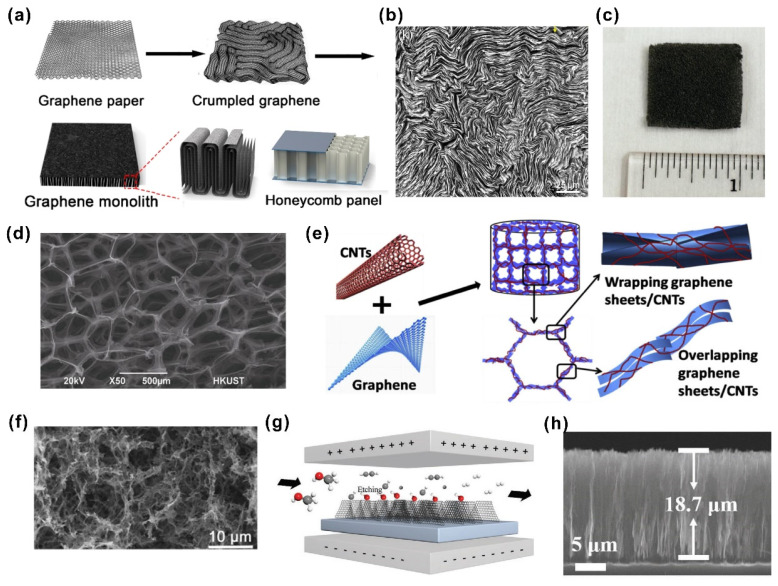
(**a**) The fabrication process for graphene monolith. (**b**) The top view SEM image of vertically aligned graphene sheets ((**a**,**b**) reprinted/adapted with permission from Ref. [81]. 2019, the American Chemical Society). (**c**) The picture of 3D graphene skeleton foam. (**d**) The SEM image that shows the 3D interconnected thermal pathways inside graphene foam ((**c**,**d**) reprinted/adapted with permission from Ref. [83]. 2014, Elsevier). (**e**) The method of super-elastic graphene–carbon nanotube aerogel. (**f**) The SEM image of as-prepared graphene–carbon nanotube aerogel ((**e**,**f**) reprinted/adapted with permission from Ref. [79]. 2016, Elsevier). (**g**) The growth of vertical graphene arrays using electrical field. (**h**) The cross-sectional SEM image of graphene arrays with a height of 18.7 µm ((**g**,**h**) reprinted/adapted with permission from Ref. [85]. 2020, Wiley).

For instance, graphene monolith [81] was fabricated with vertically aligned graphene sheets and horizontally aligned graphene layers covering the top and bottom sides by Dai et al., which appeared as honeycomb panel-like aligned microstructures (Figure 4a). The diminishment of the thermal contact resistance was due to the horizontally graphene layers on both sides. The top view SEM image in Figure 4b shows the microstructural features of compact stacking and vertical alignment between graphene monoliths. The fabricated graphene monolith displayed a metal-level thermal conductivity of 500 W·m^−1^·K^−1^ and a unique soft compression modulus as well, thus representing its potential in practical thermal management applications. Zhang et al. [83] proposed a type of 3D graphene skeleton foam with an ultralow interfacial thermal resistance (0.04 cm^2^·K^−1^·W^−1^) that was one order of magnitude lower than that of conventional TIMs (Figure 4c). The soft mechanical properties and 3D interconnected thermal pathways (as shown in Figure 4d) were essential to achieve the low interfacial thermal resistance, as well as decent thermal conductivity for high-power electronics cooling. Nevertheless, pristine graphene foams still suffered from decreased thermal transport due to inherently high porosity and low density. Lv et al. [79] investigated super-elastic graphene–carbon nanotube aerogel prepared via a hydrothermal method and freeze-drying treatment, as illustrated in Figure 4e. The simultaneous achievements of both excellent thermal conductivity (88.5 W·m^−1^·K^−1^) and low interfacial thermal resistance (0.136 cm^2^·K^−1^·W^−1^) were attributed to the entanglement interactions between CNT and graphene. In addition, the elastic properties were also greatly improved. Furthermore, there was a substantial enhancement in thermal conductivity under external pressure conditions, which boosted thermal transport capacity. In addition to graphene and other carbon materials being prepared as paper, foam and films, graphene arrays can also be assembled by electrical field manipulation. Xu et al. [85] adopted an electrical field approach to help the growth of vertical graphene arrays (Figure 4g). The critical synthetic step is to promote the growth rate and inhibit the formation of array defects in an alcohol-based carbon source atmosphere. Figure 4h shows the cross-sectional SEM image of graphene arrays with a height of 18.7 µm. A high thermal conductivity of 53.5 W·m^−1^·K^−1^ was obtained by the formation of aligned vertical graphene arrays without obvious defects. The practical heat dissipation test indicated its great potential as a heat spreader for electronics.

### 3.4. Polymer-Based Thermal Interface Materials

Polymers are commonly utilized as a matrix for TIMs because of their excellent mechanical properties, easiness to handle and unique flexibility [86,87,88,89,90]. Most current polymer TIMs, however, have lower thermal conductivity (~0.2 W·m^−1^·K^−1^) that cannot meet the demand of thermal management for modern electronics with remarkably expanded power consumption. Some researchers have introduced high thermally conductive fillers, such as metals [68,91,92], carbon nanomaterials [93,94,95] and low-dimensional inorganic materials [96,97], into polymer matrices to significantly improve overall thermal transport performances.

**Figure 5:**
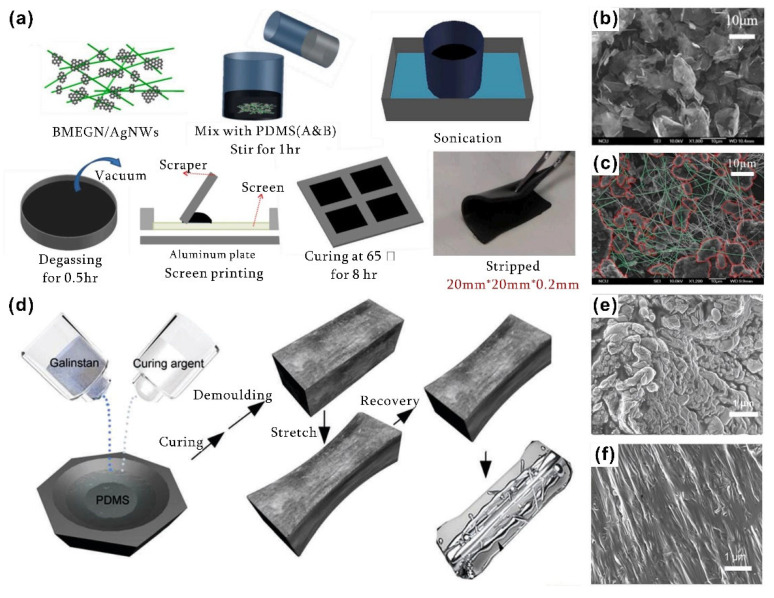
(**a**) The fabrication procedure for hybrid fillers incorporated into a PDMS matrix. (**b**) Pristine 2D graphite nanoplate filler. (**c**) The uniform dispersion of 2D graphite nanoplates with 1D silver nanowires with a 3D interconnected network in the PDMS matrix (Figure 5a–c reprinted/adapted with permission from Ref. [98]. 2020, Elsevier). (**d**) The mixing process for liquid metals with PDMS. (**e**,**f**) The micro/nanochannels of liquid metal inclusions formed with 10% and 50% deformation quantity (Figure 5d–f reprinted/adapted with permission from Ref. [99]. 2018, Royal Society of Chemistry).

Chang et al. [98] prepared PDMS-based TIMs by introducing hybrid fillers composed of graphite nanosheets and silver nanowires. The fabrication procedure is described in Figure 5a. The SEM image in Figure 5b,c confirms the uniform dispersion of 2D graphite nanoplates filler with 1D silver nanowires with a 3D interconnected network in the PDMS matrix, where the red part represents the graphite nanoplates generated via ball milling and the green line acts represents the silver nanowires. The enhanced in-plane thermal conductivity of 29.2 W·m^−1^·K^−1^ and the through-plane thermal conductivity of 4.9 W·m^−1^·K^−1^ were ascribed to the interconnection of silver nanowires and the large specific surface area of graphene sheets, respectively, leading to the lower percolation threshold. The robust flexibility of the hybrid filler–PDMS material provided an indispensable necessity for the application of heat dissipation in high-power electronics.

**Figure 6:**
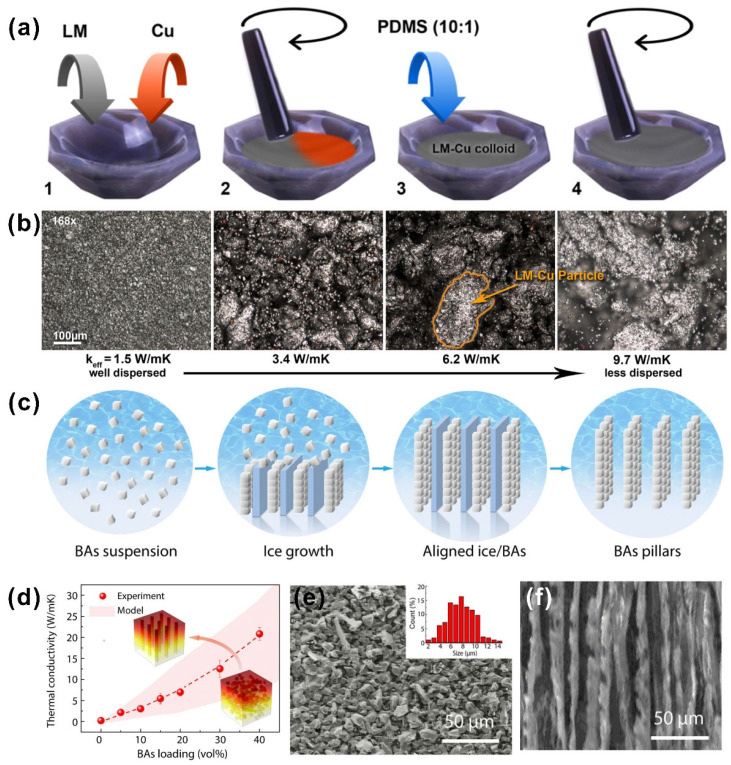
(**a**) The simple mixing method to incorporate the liquid metal and copper powder into polymer matrix. (**b**) The difference in thermal conductivity of as-prepared composite ((**a**,**b**) reprinted/adapted with permission from Ref. [100]. 2018, the American Chemical Society). (**c**) The schematic of the growth of aligned structured boron arsenide nanocolumns produced using the directional freeze-drying technique. (**d**) The thermal conductivity of as-prepared composites with different boron arsenide loadings. (**e**,**f**) The microstructural SEM image of the boron arsenide crystals and aligned boron arsenide ((**c**–**f**) reprinted/adapted with permission from Ref. [101]. 2021, Springer Nature).

Liquid metals have also been frequently incorporated into polymer to form thermal conductive paths. Zhao et al. [99] found that mixing liquid metals with PDMS could produce a TIM with both a low thermal resistance and a high elasticity (Figure 5d). Moreover, the liquid metal inclusions could form micro/nanochannels under applied stress, resulting in a large increase in the lateral thermal conductivity of 8.3 W·m^−1^·K^−1^, as shown in Figure 5e,f (10% deformation of liquid metal on the top and 50% deformation on the bottom). Heat dissipation experiments conducted on a smartphone demonstrated the efficient thermal management of liquid metal–PDMS composites. To further optimize the thermal performance, Ralphs and his group members [100] used a simple mixing method to incorporate the liquid metal and copper powder into the polymer matrix (Figure 6a). Liquid metal composite fillers could be fixed throughout in situ alloying of gallium with copper, thus preventing leakage and contamination of liquid metals. Figure 6b reveals the difference in the thermal conductivity of the as-prepared composite, where the fillers ranged from uniformly dispersed to highly poly-dispersed in the matrix, indicating that the formation of the connection between large fillers inside the polymer could promote the overall thermal performance.

Recently, Cui et al. [101] reported a self-assembled boron arsenide and epoxy advanced thermal interface composite based on the ice template process. Directional freezing and subsequent freeze-drying techniques (Figure 6c) were used to build highly aligned structured boron arsenide nanocolumns in resin with a superb thermal conductivity of up to 21 W·m^−1^·K^−1^ (Figure 6d). The microstructural SEM images of the boron arsenide crystals and aligned boron arsenide are shown in Figure 6e,f. The softness of the product allowed high-quality conformal contact with electronic packaging. As an emerging boron compound semiconductor, cubic boron arsenide, with a thermal conductivity that is several times higher than that of common metallic thermal conductors (e.g., more than three times the thermal conductivity of copper) is expected to be explored for future advanced thermal management applications.

**Figure 7:**
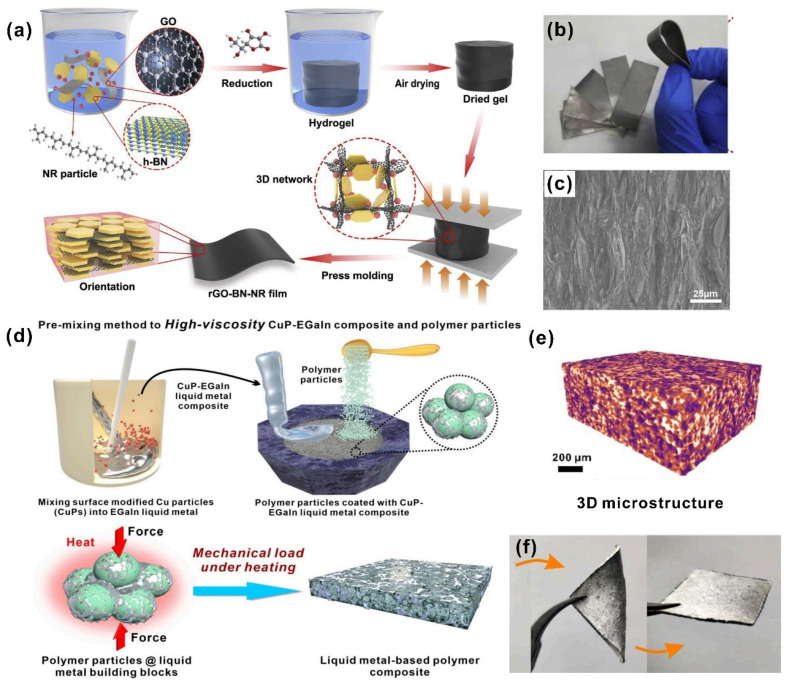
(**a**) The schematic of the fabricating procedure for the rGO–BN–rubber composite. (**b**) The photograph of the obtained films. (**c**) The SEM image of the obtained films under pressure ((**a**–**c**) reprinted/adapted with permission from Ref. [102]. 2020, Elsevier). (**d**) Schematic illustration of the hybrid filler (pre-mixed copper and liquid metal) flowing to fill the gaps between TPE particles under pressure load during heating. (**e**) The 3D microstructure network of liquid metals as produced by a microcomputed tomography (Micro-CT) experiment. (**f**) The optical images of liquid-metal-based TPE composite (bending and after bending) ((**d**–**f**) reprinted/adapted with permission from Ref. [92]. 2022, Elsevier).

It is crucial for the thermally conductive fillers to assemble a conductive network in polymer matrixes. Mechanical sintering is an emerging and simple way to accomplish this requirement. For example, Li et al. [102] developed a novel GO-assisted gelation and hot-pressing method to build an rGO–BN–rubber composite, as depicted in Figure 7a. A photograph and SEM image of the obtained films are shown in Figure 7b,c, where the aligned rGO and BN interconnected network was employed under pressure. The prepared multifunctional film displayed a high thermal conductivity of 16.0 W·m^−1^·K^−1^, good flame retardancy and antistatic properties. Such mechanical loading with heat motivated not only solid fillers such as BN, rGO, etc., to form thermal conductive pathways in the polymer matrix, but also encouraged liquid fillers, such as liquid metal semi-liquid/liquid, to construct oriented microstructures. In our previous work [92], the fluidic liquid metal fillers were pre-mixed with TPE powder to construct an isotropic thermal conduction network under mechanical loading with heat. The pre-mixed hybrid filler of copper and liquid metal could flow to fill the gaps between TPE particles under pressure load during heating (Figure 7d), and became more compact with a thermal conductivity of up to 32.7 W·m^−1^·K^−1^ and an electrical conductivity of 1.18 × 10^6^ S·m^−1^. Additionally, there was no leakage under external pressure (1.5 MPa), which is almost the maximum pressure for TIM encapsulation. A microcomputed tomography (Micro-CT) experiment was conducted to demonstrate the 3D microstructure network of liquid metals (Figure 7e), with the yellowish portion representing the continuous network of liquid metal hybrid fillers in the TPE matrix. A TPE elastomer and soft liquid metal contributed to the fantastic elasticity and deformability of the liquid-metal-based polymer composite, which could be folded several times without damage (Figure 7f). The cost of raw materials was also an issue that must be considered for advanced TIMs in the marketplace. Their low cost and ease of handling make liquid-metal-based TPE materials highly competitive. This work also provides a new strategy for constructing isotropic networks of a fluidic liquid filler under mechanical sintering.

In summary (see Table 2), metal-based TIMs have high thermal conductivity and a low coefficient of expansion and are easy to apply. However, metal-based TIMs are easily oxidized and may form intermetallic compounds with high thermal conductive fillers during application, thus generating air voids between the interfaces and hindering the heat dissipation. Carbon-based TIMs exhibit high thermal conductivity and low thermal contact resistance, no curing, no leakage and good mechanical properties, but further work is still needed to solve carbon-based materials’ processability and dispersibility issues. Polymer-based TIMs have excellent dielectric properties, low density, low cost and no leakage and are easy to handle, but they have a lower thermal conductivity than carbon-based and metal-based composites. A polymer-based composite would become brittle and hard with the addition of thermal conductive fillers. In addition, some of the polymer matrixes need to be cured, and their long-term stability under thermal cycling is also not negligible.

### 3.5. Methods of Thermal Measurement for Advanced Thermal Interface Materials

The methods of thermal measurement for TIMs can be generally divided into steady-state and nonsteady-state (transient) methods at the macroscopic level. As for the nanoscale, thermal measurements techniques, such as scanning thermal microscopy (SThM) [105], the time-domain thermoreflectance method (TDTR) [106] and the Raman optothermal technique [107], are normally used to study and measure nanoscale thin films. Scanning thermal microscopy (SThM) was employed by Assy et al. [108] to measure the interfacial heat transfer at the nanoscale between different samples. The probe under vacuum could be used to explore the heat transfer through the nanoscale point contacts. The local temperature pattern of samples with nanoscale resolution could be identified to investigate the heat transfer behavior in the sample. Therefore, it is an important tool for the thermal analysis of nanomaterials. However, the impact of the native oxide layer on the probe surface regarding the real chemistry and roughness is still a pressing issue in SThM measurement. Zhao et al. [109] obtained the thermal conductivity of (Ti,Al)N ceramic coatings using the time-domain thermoreflectance (TDTR) method. The TDTR method could accurately manipulate the association between reflectance and temperature for thermal measurement of nanomaterials. However, the accuracy of the obtained thermal conductivity value depends on the thermal properties of substrate, pulse width and repetition rate. In Limbu’s previous work [110], the room temperature thermal conductivity of polycrystalline twisted bilayer graphene was measured based on the Raman optothermal technique. The Raman optothermal technique is a noncontact type of thermal measurement that avoids the influence of the substrate on the measured thermal conductivity of the sample. Nevertheless, the accuracy of the Raman optothermal technique is notably affected by the laser spot size, optical absorption of sample, contact area, etc., and further research is needed to advance the measurement accuracy.

In this review, we focus on the steady-state and transient methods of thermal measurements for microscale advanced TIMs for high-power electronics.

The steady-state method for measuring thermal conductivity and thermal contact resistance is based on the ASTM-D5470 standard [79]. The sample is placed between the upper and lower plates (cold and hot plates) to simulate the actual heat transfer. According to Fourier’s law, thermal conductivity can be derived by the obtained apparent temperature gradient in the steady state [92,98]. In the practical steady-state method measurement based on the ASTM-D5470 standard, measuring the thermal conductivity and thermal contact resistance is achieved through acquiring the fitted linear relationship between the total thermal resistance and thickness of the same material. The reciprocal of the slope of the fit line is the thermal conductivity of the sample, and the value of thermal resistance on the y axis is the thermal contact resistance of the sample when the thickness is zero [79,92]. The steady-state method is easy to use at the operating temperature, and can effectively measure the thermal conductivity and thermal contact resistance directly. However, for those samples with low thermal contact resistance, it is difficult to ensure the accuracy of the measurement system.

Recently, nonsteady-state measurement techniques have been developed rapidly, including laser flash analysis (LFA) [111] and the hot-wire method [60]. Among them, LFA measurement is widely utilized due to its fast response and simple operation. The method is performed by emitting laser pulses, which are absorbed by the sample, and the time profile of temperature changes on the other side of sample is detected and recorded via an infrared detector. The thermal diffusivity (α = 0.1388 × d^2^/t_1/2_) can be calculated from the collected half-rise time (t_1/2_) and the thickness of the sample (d). The thermal conductivities of samples were calculated by [61]

λ = α × ρ × C_p_, (5)

where *α* is the thermal diffusivity of the material, and ρ and C_p_ are the density and specific heat capacity of sample, respectively. For the thermal resistance measurement of samples based on the transient method, Zhao et al. [112] demonstrated the thermal resistance measurement of a 3D graphene foam–PDMS composite using laser flash analysis (LFA). The thermal diffusion coefficient of the bulk sample could be measured directly through LFA, and the thermal contact resistance between the sample and copper substrate could be calculated by the designed sandwich model, where the sample is sandwiched between two smooth copper substrates. According to the proposed assumptions, the thermal contact resistance could be obtained, and the feasibility of LFA measurement in practical applications was demonstrated. The LFA can achieve the measurement of batch samples at one time with a fast response and easy handling, but the thermal conductivities cannot be directly obtained, and further calculations are required.

Nowadays, the development of thermal measurement techniques must constantly keep pace with the development of materials to achieve accurate measurement. The appropriate choice of thermal measurements should rely on the intrinsic properties of the sample and the actual measurement requirements.

## 4. Overview and Future Prospects

This review gives a brief introduction of the advanced TIMs used in heat dissipation of high-power electronics. High-power electronics generate a large amount of heat per unit time and are usually used under a relatively high temperature. Therefore, micro- and nanoscale TIMs for high-power electronics are required to possess not only high thermal conductivity and low interfacial thermal resistance, but also superb thermal and chemical stabilities and good compression properties. In this review, the typical thermal conduction models of TIMs have been discussed, and thermal performances of different composite materials for high-power electronics have been introduced in detail. Advanced metal-based and carbon-based TIMs have been highly regarded as high-quality TIMs with superb high thermal conductivity for heat conduction and strong thermal and chemical stabilities in high-power electronics since the matrix materials themselves have high thermal conductivity, high decomposition temperature and chemical inertness compared to the organic matrix. As for metal-based TIMs, liquid metal composites can fill the air gap fully as they have unique high fluidity, and they can form intermetallic compounds with metallic fillers, which helps reduce the thermal contact resistance. Lightweight carbon-based TIMs also exhibit high thermal conductivity and thermal stability, and are therefore more suitable for heat dissipation in aerospace-related applications. As for polymer-based TIMs, although polymers exhibit a relatively lower decomposition temperature, their high flexibility enables their wide use in a variety of situations. The low thermal conductivity of the matrix can be improved by mixing highly thermal conductive fillers into the matrix. Increasing the number of those fillers can create heat conduct pathways and networks in the structure and effectively improve the thermal conductivity.

The miniaturization and integration of electronic devices require higher power density heat dissipation. Herein, we introduce new issues found in developing advanced TIMs to promote the heat dissipation of high-power devices. (1) To further improve the heat dissipation ability of TIMs, increasing thermal conductivity and reducing interfacial thermal resistance are of vital importance. Novel matrices, filler materials and preparation methods need to be applied to increase the thermal conductivity of TIMs. High-melting-point metals such as silver, copper and aluminum have high thermal conductivity but cannot fill air gaps precisely, which results in a relatively high interfacial thermal resistance of TIMs. As a result, it is possible to integrate liquid metal or carbon-based materials with high-melting-point metals to reduce the interfacial thermal resistance. Along with carbon-based materials, other low-dimensional inorganic materials with high thermal conductivity such as boron arsenide have great potential for the development of advanced TIMs. Surface modification of filler particles and formation of highly thermal conductive intermetallic compounds will reduce the interfacial thermal resistance. (2) The service life of TIMs needs to be further improved to support their utilization for a long period of time without loss of heat dissipation performance. Suitable TIMs need to have long-term stability, oxidation resistance, antifatigue performance and other abilities. (3) For high-power electronics, the thermal and chemical stability of TIMs need to be enhanced since the temperature of the working chip in the unit area and unit time is much higher than in regular electronics. (4) The cost of TIMs is still relatively high, and they need proper utilization and recycling to reduce electronic waste. This review briefly summarizes the recent progress in research on advanced TIMs for high-power electronic devices, which is helpful for designing the structure of high-power electronic devices and selecting suitable TIMs for their heat dissipation.

## Figures and Tables

**Table 1:** Typical thermal conductivity models of composite-based TIMs.

Model	Formula	Remarks	Reference
Series	${\lambda =\mathrm{Vk}}_{2}+\left(1-V\right)k_{1}$	Polymer is arranged in parallel to thermal flux.	[45,46]
Parallel	${1/k=V/k}_{2}\;+\;\left(1-V\right){/k}_{1}$	Polymer is arranged in the direction of thermal flux.	
Agari and Uno	${\mathrm{logk}=\mathrm{VC}}_{2}{\mathrm{logk}}_{2}{+(1-V)\log(C}_{1}k_{1})$	Fillers are randomly, isotropically dispersed in a thermal conduction system based on the generalization of parallel and series conduction models.	
Maxwell	$k_{\mathrm{eff}}=\frac{{2k}_{f}{\;+\;k}_{s}\;+\;2\emptyset_{s}\left(k_{s}{-k}_{f}\right)}{{2k}_{f}{\;+\;k}_{s}-\emptyset_{s}\left(k_{s}{-k}_{f}\right)}\cdot k_{f}$	Fillers as noninteracting homogeneous spheres are randomly distributed. It is only valid in low volume fractions of fillers.	[30,31]
Hasselman and Johnson	$k_{\mathrm{eff}}{=k}_{m}\frac{\left[2\left(\frac{k_{d}}{k_{m}}-\frac{k_{d}}{{\mathrm{ah}}_{c}}-1\right)V_{d}+\frac{k_{d}}{k_{m}}\;+\;\frac{{2k}_{d}}{{\mathrm{ah}}_{c}}+2\right]}{\left[\left(1-\frac{k_{d}}{k_{m}}+\frac{k_{d}}{{\mathrm{ah}}_{c}}\right)V_{d}\;+\;\frac{k_{d}}{k_{m}}\;+\;\frac{{2k}_{d}}{{\mathrm{ah}}_{c}}\;+\;2\right]}$	Based on the Maxwell model, this model considers the influence of the size of fillers and the interfacial thermal resistance.	[32,34]
Bruggeman	${1-V}_{f}=\frac{\lambda_{f}{-\lambda }_{c}}{\lambda_{f}{-\lambda }_{p}}{\left(\frac{\lambda_{p}}{\lambda_{c}}\right)}^{\frac{1}{3}}$	The model considers dilute suspensions of spheres in a homogeneous medium, and can be applied for relatively high volume fractions of fillers.	[36,37]
Eshelby	$k_{\mathrm{eff}}{=k}_{0}{\;+\;c}_{1}\left(K_{1}{-K}_{0}\right)T$	The fillers do not interact with each other. It is only valid for low volume fractions of fillers.	[39,47]
Mori–Tanaka	$k_{\mathrm{eff}}=\left(c_{0}k_{0}{\;+\;c}_{1}k_{1}T\right){\left(c_{0}{I+c}_{1}T\right)}^{-1}$	The model considers interactions between fillers. It is valid for up to 20% volume fractions of fillers.	
Tavangar	$k_{c}{=k}_{m}\frac{\frac{k_{d}^{\mathrm{eff}}}{k_{m}}\left({1\;+\;2v}_{d}\right)\;+\;\left({2-2v}_{d}\right)}{\frac{k_{d}^{\mathrm{eff}}}{k_{m}}\left({1-v}_{d}\right)\;+\;\left({2\;+\;v}_{d}\right)}$	The model is accurate at high volume fractions of fillers and can be applied for more than two components.	[48,49]
Hamilton and Crosser	${k=k}_{1}\left[\frac{k_{2}\;+\;\left(n-1\right)k_{1}-\left(n-1\right)V_{2}{(k}_{1}{-k}_{2})}{k_{2}\;+\;\left(n-1\right)k_{1}{\;+\;V}_{2}{(k}_{1}{-k}_{2})}\right]$	The model considers the shape effect of the particles.	[50]
Lewis and Nielsen	$\frac{k_{c}}{k_{m}}=\frac{1\;+\;\mathrm{AB}\emptyset_{2}}{1-B\psi \emptyset_{2}}$	The model considers the shape effect of the particles and the orientation of packing for a two-phase system.	[40,51]
Cheng and Vachon	$\frac{1}{k_{m}}=\frac{1}{\sqrt{\left\{C\left(k_{c}{-k}_{d}\right)\left[k_{c}{\;+\;B(k}_{d}{-k}_{c})\right]\right\}}}\ln\frac{\sqrt{k_{c}\;+\;B\left(k_{d}{-k}_{c}\right)}\;+\;\frac{B}{2}\sqrt{C\left(k_{c}{-k}_{d}\right)}}{\sqrt{k_{c}\;+\;B\left(k_{d}{-k}_{c}\right)}-\frac{B}{2}\sqrt{C\left(k_{c}{-k}_{d}\right)}}\;+\;\frac{1-B}{k_{c}}$	The model can be used to predict the thermal conductivity of heterogeneous mixtures.	[52,53]
Percolation theory model (PTM)	${k=k}_{p}{\left(\frac{k_{c}}{k_{p}}\right)}^{{\left(\frac{1-\emptyset }{1-\emptyset_{c}}\right)}^{n}}$	The model considers the filler shape and size distribution.	[38,41]

**Table 2:** A summary of fabrication methods and thermal performances of recently developed advanced thermal interface materials.

Materials		Fabrication Method	Thermal Conductivity (W·m^−1^·K^−1^)	Thermal Measurement Method	Year [Ref.]
Metal- based TIMs	Silver nanoparticles	Bimodal sintering	278.5	LFA	2015 [65]
	Silver paste	Pressure-less low-temperature sintering	354	Transient thermal measurement system (self-built)	2016 [64]
	Cu-graphite-Cu	Electroplating	526–626	LFA	2016 [59]
	EGaIn/Cu	Electrical-polarization-assisted	50	Hot disk TPS	2017 [72]
	GaInSn/W	Oxide-layer-assisted mixing	62 ± 2.28	Hot disk TPS	2019 [60]
	Ga/rGO	Mechanical mixing+ oscillating ball mill	126 (parallel) 10.5 (perpendicular)	LFA	2021 [70]
	Ga/graphite@ Ni	Planetary ball mill	44.6	LFA	2021 [61]
	EGaIn/Cu@ CPTES	Molecule thermal linker	65.9	LFA	2021 [62]
	Ga/diamond/carbon fiber	Repeated compression	129	LFA	2022 [63]
Carbon- based TIMs	Graphene	Optimized mixture of graphene and multilayer graphene	14	LFA	2012 [23]
	Graphene/CNT aerogel	hydrothermal method and freeze-drying	88.5	ASTM D5470	2016 [79]
	Graphene monolith	Mechanical machining process	143	LFA	2019 [81]
	Graphene array	Chemical vapor deposition	53.5	TDTR	2020 [85]
	Graphene paper	Rapid filtration	12.6	LFA	2021 [103]
Polymer- based TIMs	PDMS/GaInSn	Mixing and stretching	8.3	Thermal meter (DRL-Ⅲ)	2018 [99]
	PDMS/GaInSn/Cu	In situ alloying	17	ASTM D5470	2018 [100]
	PVDF/BNNS	Electrospinning	16.3	LFA	2019 [96]
	PVA/BNNS	Electrostatic spraying	21.4	LFA	2019 [97]
	Ag@Cellulose/Al_2_O_3_/graphene	Vacuum-assisted self-assembled	9.09	LFA	2020 [104]
	Rubber/rGO/BN	Gelation and hot compression	16.0	LFA	2020 [102]
	PDMS/graphite/AgNWs	Simple mixing	29.2	LW-9389	2020 [98]
	Epoxy–boron arsenide	Ice-templated self-assembled	21	LFA	2021 [101]
	TPE/EGaIn/Cu	Hot pressing	32.71	LW9389	2022 [92]

## Data Availability

Not applicable.
